# Mutations on *ent*-kaurene oxidase 1 encoding gene attenuate its enzyme activity of catalyzing the reaction from *ent*-kaurene to *ent*-kaurenoic acid and lead to delayed germination in rice

**DOI:** 10.1371/journal.pgen.1008562

**Published:** 2020-01-10

**Authors:** Hui Zhang, Ming Li, Dongli He, Kun Wang, Pingfang Yang

**Affiliations:** 1 State Key Laboratory of Biocatalysis and Enzyme Engineering, School of Life Sciences, Hubei University, Wuhan, China; 2 National Key Laboratory of Crop Genetic Improvement and National Centre of Plant Gene Research (Wuhan), Huazhong Agricultural University, Wuhan, China; 3 School of Life Sciences, Wuhan University, Wuhan, China; Chinese Academy of Sciences, CHINA

## Abstract

Rice seed germination is a critical step that determines its entire life circle, with seeds failing to germinate or pre-harvest sprouting both reduce grain yield. Nevertheless, the mechanisms underlying this complex biological event remain unclear. Previously, gibberellin has been shown to promote seed germination. In this study, a delayed seed germination rice mutant was obtained through screening of the EMS induced mutants. Besides of delayed germination, it also shows semi-dwarfism phenotype, which could be recovered by exogenous GA. Through re-sequencing on the mutant, wild-type and their F_2_ populations, we identified two continuous mutated sites on *ent-kaurene oxidase 1* (*OsKO1*) gene, which result in the conversion from Thr to Met in the cytochrome P450 domain. Genetic complementary analysis and enzyme assay verified that the mutations in *OsKO1* gene block the biosynthesis of GA and result in the defect phenotypes. Further analyses proved that OsKO1 could catalyze the reaction from *ent*-kaurene into *ent*-kaurenoic acid in GA biosynthesis mainly at seed germination and seedling stages, and the mutations decrease its activity to catalyze the step from e*nt*-kaurenol to *ent*-kaurenoic acid in this reaction. Transcriptomic and proteomic data indicate that the defect on GA biosynthesis decreases its ability to mobilize starch and attenuate ABA signaling, therefore delay the germination process. The results provide some new insights into both GA biosynthesis and seed germination regulatory pathway in rice.

## Author summary

Rice is propagated through seeds. Synchronized seed germination upon the stimuli of exogenous environmental factors is critical for its production. The exogenous factors are mediated by endogenous factors, including gibberellins. It will help to obtain more comprehensive knowledge about the regulation of seed germination to explore the genes involved in biosynthesis and signaling of gibberellins. We found mutations on *ent*-*kaurene oxidase* 1 gene lead to the defect of gibberellin biosynthesis, and thus the delayed germination in rice. Through genetic and biochemical analyses, we proved that ent-kaurene oxidase 1 is a new enzyme catalyzing the reaction in the gibberellins biosynthetic pathway other than ent-kaurene oxidase 2 in rice. We also showed that it specifically functions at seed germination and seedling stages. The results provide strong evidence for the enzyme activity and catalyzing features of ent-kaurene oxidase 1 in rice. The pathway through which gibberellins regulate rice seed germination is partially discovered. However, the regulation on the gibberellin biosynthesis during germination still needs further studies.

**Highlights**

1. Strong evidences at both genetic and biochemical levels proved the function of *OsKO1* gene in GA biosynthesis;
2. *OsKO1* gene might function mainly at seed germination and young seedling stages;
3. Different steps of the reaction from ent-kaurene to ent-kaurenol, and then to ent-kaurenoic acid in GA biosynthetic pathway might be catalyzed by different motifs of ent-kaurene oxidase in the intact enzyme context.

## Introduction

Rice is one of the main cereal grains that feed the world population. Rice seed with its independent role as a new generation could provide itself with enough reserves until the establishment of new seedling [1]. Defect of seed germination at either rate or timing could impair grain yield in agriculture [2]. It is vital to disclose the molecular mechanisms that regulate rice seed germination, which might help to facilitate molecular design in rice breeding.

Seed germination, which starts with water absorption by the dry seed and ends at radicle protrusion, is controlled by the crosstalk among various phytohormones and environmental stimuli. The phytohormones include abscisic acid (ABA), gibberellic acid (GA), ethylene, brassinosteroids, salicylic acid, cytokinin, auxin, jasmonic acid and oxylipins, with the first two ABA and GA playing the major roles. ABA controls storage reserves accumulation, and induces the expression of late embryogenesis abundant proteins (LEAs) that accumulated during seed maturation to protect seeds against desiccation. ABA also inhibits the germination of seeds on mother plant [3]. Considerable evidences have proven that ABA functions in dormancy maintenance [4–6], in which pre-harvest sprouting mutants in rice (*Oryza sativa* L.) all show reduced ABA content [7]. Based on the studies in *Arabidopsis*, it is known that series transcription factors form a cascade in ABA signaling pathway to control seed germination in which ABI1 and ABI2 are two homologous phosphatases that negatively regulate *ABI3*. ABI3 is a transcription activator that will be degraded by the recognition of AIP2, an E3 ligase ABI3 interaction protein [8–10]. WRKY41 directly activates ABI3 together with ABA to maintain dormancy [11]. ABI5 is a bZIP transcription factor that functions at the downstream of ABI3 to arrest seed germination [12]. ABI4 contained an APETALA2 (AP2) DNA binding domain in a separate pathway to restrain germination [13].

In contrast to ABA, GA functions in promoting germination and radical protrusion. GA induces the expression of enzymes that mobilize storage reserves like starch, proteins, and lipids [14]. Rice delayed germination mutants show reduced expression of either GA synthetic genes or GA responsive genes or both [8,15]. In rice, GA functions through activating the expression of *α-amylase* in aleurone layer to degrade starch into soluble sugars in endosperm, and the soluble sugars in endosperm are transported into embryo [16]. Reduced germination rate and seedling growth are always accompanied by decreased expression of *α-amylase* [17,18]. Mutants with reduced germination rate and delayed germination phenotypes all show the repression of *α-amylase* genes [8,19]. Recently characterized rice germination delayed mutants include germination defective 1 (*GD*1), knock-down mutant of rice lectin receptor-like kinase (*OslecRK*), and *Oryza sativa* delayed seed germination 1 (*OsDSG*1). GD1 is B3 domain containing repressor, and OsDSG1 is a ring finger E3 ligase. Their delayed germination phenotype accompanied with reduced plant height, decreased endogenous GA levels, reduced expression of *α-amylase* genes, down-regulated GA biosynthetic genes and up-regulated GA catabolic genes [8,15,19]. Especially an endosperm-imposed dormancy prolonged mutant seed dormancy1-2 (qSD1-2) has mutation on OsGA20ox2 was observed with semi-dwarf plant, decreased GA levels, extended dehydration and seed maturity [20]. Taken together, the control of seed germination relies on the fine-tuning of GA and ABA levels.

Seven genes have been shown to be involved in the biosynthesis of active GAs such as GA_1_, GA_3_, and GA_4_. Four single genes include *ent*-copalyl diphosphate synthase (*CPS*), *ent*-kaurene synthase (*KS*), *ent*-kaurene oxidase (*KO*, *CYP701A*), *ent*-kaurenoic acid oxidase (*KAO*, *CYP88A*) catalyze early steps of GA biosynthesis from precursor geranylgeranyl diphosphate (GGDP) to GA_12_. Two terpene synthases CPS and KS catalyze the conversion of GGDP to *ent*-kaurene on plastid [21,22] followed by two cytochrome P450 monooxygenases *KO* and *KAO*. *KO* converts *ent*-kaurene to *ent*-kaurenoic acid, and *KAO* catalyzes the formation of GA_12_ from *ent*-kaurenoic acid [23,24]. GA_12_ is the common precursor of active GA_1_ and GA_4_, while GA 13-oxidases are needed in the converting of GA_12_ into GA_53_ from 13-hydroxylation pathway. These are followed by the conversion of GA_53_ into GA_1_ in 13-hydroxylation pathway and the conversion of GA_12_ to GA_4_ in the non-13-hydroxylation pathway catalyzed by GA 20-oxidase (*GA20ox*) and GA 3-oxidase (*GA3ox*) that with small gene families [25]. Various mutants of the seven genes that show dwarfism phenotype have been isolated. There are five *OsKO* genes which are contiguously arranged in a 120 kb highly linked region on the sixth chromosome (**S1A Fig**). Previous study has shown that *OsKO2* is the right gene encoding the correct *ent*-kaurene oxidase in GA biosynthetic pathway in rice [26,27]. Their study showed that another *OsKO* (It is named as *OsKO1* in this study, but updated as *OsKO5* in the newly released rice genome annotation version; **S1A Fig**) gene might also involve in GA biosynthesis [26]. OsKO2 is a Cytochrome P450 monooxygenase that may locate on the outer membrane of plastid [22]. The kinesin-like protein GDD1 binds to the promoter of *OsKO2* and activates its expression to maintain endogenous GA levels in rice [28]. Recently, a study showed that both OsKO1 and OsKO5 might also involve in the GA biosynthesis in cooperation with OsKO2 based on the phenotyping of the corresponding gene’s CRISPR/Cas9 edited rice lines, of which both *OsKO1* and *OsKO5* edited lines were dwarfism [29]. However, no further direct evidences verified their involvement in GA biosynthesis.

To further explore the regulatory mechanisms underlying rice seed germination, we screened a rice mutant showing both delayed germination and semi-dwarfism phenotype. MutMap sequencing of two F_2_ progeny bulks determined two SNP changes on the eighth exon of *OsKO1* gene resulting in a Thr to Met transition. Further biochemical and molecular genetic analyses proved that OsKO1 could function as well as OsKO2 in the catalyzing of *ent*-kaurene into ent-kaurenoic acid in GA biosynthesis during germination and early seedling growth in rice. The defect on this gene leads to the decreasing of GA content, and then disorder the balance between GA and ABA signaling, which delays seed germination in rice. Our results may help to obtain further understanding about both GA biosynthesis and its regulation on seed germination in rice.

## Results

### Phenotypic characterization of the germination delayed dwarfism mutant

**Fig 1:**
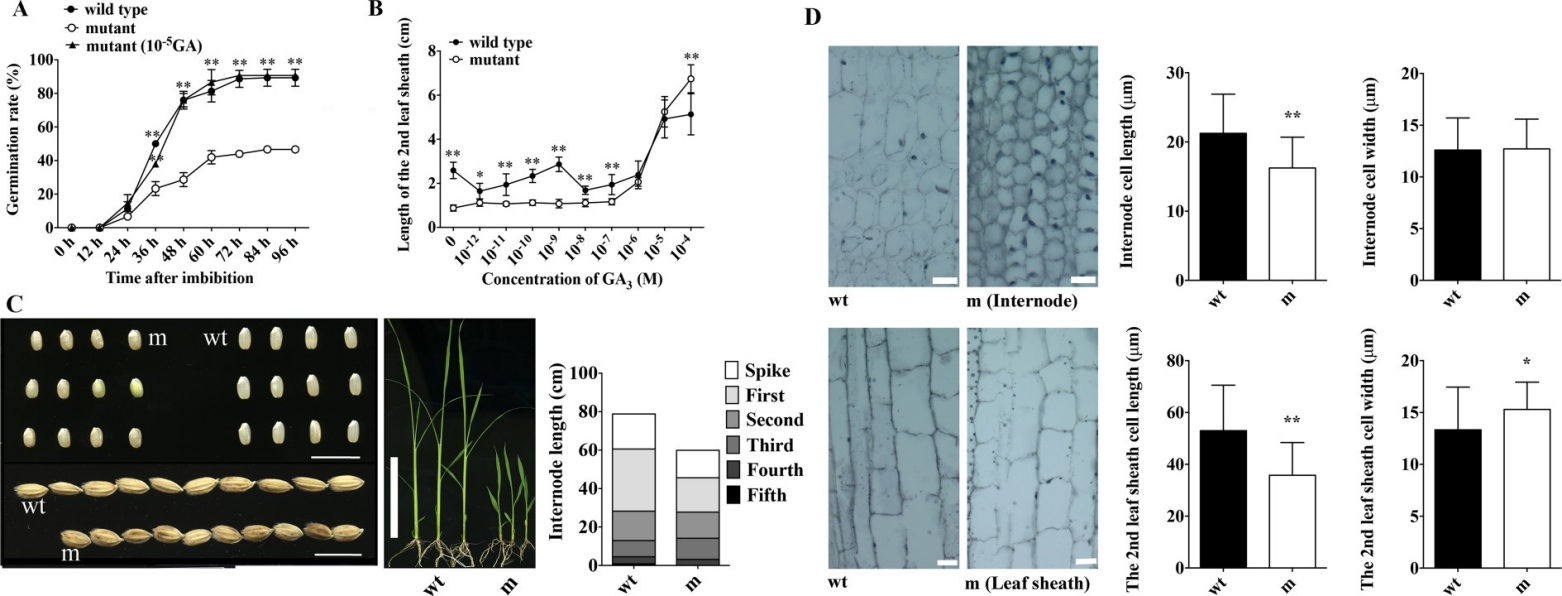
Phenotypic characterization of the mutant. (A) Comparison of seed germination among wild-type, mutant, and mutant treated with 10^−5^ M GA_3_. (B) Elongation of the second leaf sheath of mutant and wild-type rice in responding to gradient concentration of GAs. (C) Morphological phenotypes of mutant in comparison with wild type. Left and middle panels show the seed size and seedlings, respectively; and the right panel shows the comparison of different internodes. Data are means from n≥25 plants. Scale bars = 1 cm. (D) Microscope images (left panels) and quantitative analysis (right panels) of the second internode (upside panels) and leaf sheath (bottom panels) cell length. “m” stands for the mutant, and “wt” stands for the wild type. Data are means±SE from at least 4 plant sections with at least 6 cells in each section. Significant difference was determined by Student’s t-test (*p < 0.05, **p < 0.01). Scale bars = 10 μm.

Through screening the EMS mutagenesis mutants with Nipponbare background, a rice seed germination delayed mutant was obtained. To further characterize the phenotype, we reproduced it for four years, and conducted germination assay along with the Nipponbare wild-type to see its stable inheritance. The mutant seeds germinated much slower than wild-type with a 45% decrease in germination rate (**Fig 1A**). Obvious difference on the germination appeared at 36 h after imbibition (**Fig 1A**). Besides of the delayed germination phenotype, mutant also displayed smaller seeds, dwarfism, and dark green leaves (**Fig 1A and 1C**). Further studies through microscopy analysis showed that the dwarfism of mutant is resulted from its reduced cell size in internode (n ≥ 20, *p* = 0.002) and leaf sheath (n ≥ 20, *p* = 3.7 × 10^−5^) (**Fig 1C**). All these are typical GA related phenotype, which indicates that it may be a GA deficient or insensitive mutant based on previous studies [30–33].

Therefore, we treated the mutant with exogenous GA_3_ to detect it response. Similar with previous report [28], the length of second leaf sheath of mutant was comparable with that of wild-type only with up to 10^−6^–10^−5^ M GA_3_ treatment (**Fig 1B**) [28]. Then, the mutant seeds were germinated with the presence of 10^−5^ M GA_3_, which showed almost the same germination speed and rate with the wild-type (**Fig 1A**). These results showed that the mutant phenotype could be rescued by exogenous GA_3_, and it is GA responsive (**Fig 1A and 1B**). Altogether, it may be a GA deficiency mutant.

### Cloning and analyses of the mutated genes through re-sequencing strategy

To verify our hypothesis and further understand the mechanism underlying the delayed germination phenotype of the mutant, we applied whole-genome resequencing and mutmap method to characterize the mutations. The mutant was crossed with Nipponbare wild-type. All individuals of F_1_ population showed the same phenotype as the wild-type, which indicates that this is a homozygous recessive mutant. In F_2_ population, we obtained 5342 normal and 798 dwarf individuals showing a segregation ratio of 6.7:1. Considering the fact that there is 45% decrease of germination rate in the mutant, the ratio could be normalized to 3.69:1, indicating the mutant might be caused by a single gene or locus.

**Fig 2:**
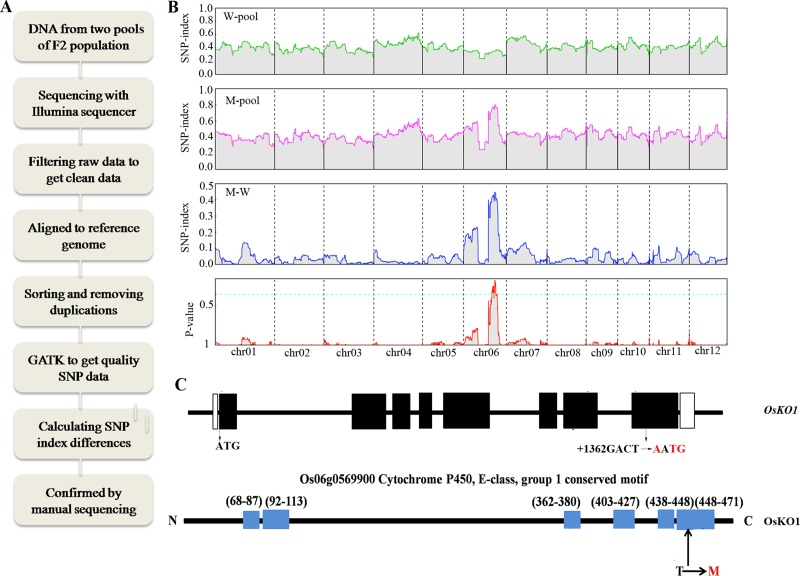
Characterization of the mutations through whole-genome resequencing and mutmap methods. (A) flowchart of the sequencing-based method. (B) Whole-genome resequencing of mutant and Nipponbare wild-type segregant F2 population to get casual mutations. Scrolling window plots show SNP index of sequencing results from wild-type pool, mutant pool and SNP index differences with their p values by chi-squared test and fisher test on 12 chromosomes. (C) Structure and mutation sites of *OsKO1* gene and its corresponding protein. Black boxes indicate exons, white boxes are UTRs, red fonts are mutated nucleotids and corresponding amino acids.

As described in M&M, two bulks of DNA from 60 individuals with mutant phenotype and 60 individuals with wild-type phenotype of F_2_ progeny were sequenced separately. Delta index analysis of the sequencing data showed a peak in a 3.05 Mb region on chromosome six (**Fig 2**), in which there are 20 genes including three *OsKO* genes, *OsKO1*, *OsKO2* and *OsKO5* (**S1 Table**). Since we have known that it might be a GA deficient mutant, we then sequenced these three *OsKO* genes including 2 kb sequence in their promoter regions again. The sequencing results were compared with those of the wild-type from RAP-DB. A short sequence replacement of ‘GACT’ to ‘AATG’ was found in the eighth exon of *OsKO1* (starting from +1362) (**Fig 2C**) without any other detected mutations in the other two *OsKO* genes. The G to A replacement results in a silent mutation, while the CT to TG replacement leads to a Thr to Met missense mutation (**Fig 2C**). To confirm the mutation, Taqman probe with 5’ labeled with FAM fluorescent group and 3’ end labeled with BHQ-1 quenching group that completely matched with 18 bp region on mutant CDS containing three mutations (AATG genotype) was used to check the linkage of the mutations to mutant phenotype. The selected 625 mutant individuals in F_2_ population all matched with probe (AATG genotype), while wild-type individuals (GACT genotype) of the parental lines all did not match with probe, which verified the tight linkage between the mutation and phenotype (**Table 1**).

**Table 1:** Taqman probe detection of linkage between SNP mutations and the mutant phenotype.

Genotype (Fluorescent signal)	Pwt: 8	Pm: 8	F_2_m: 625	F_2_wt: 63
**AATG (High peak individuals)**	0	8	625	0
**GACT/AATG (Medium-high peak individuals)**	0	0	0	35
**GACT (Undetected individuals)**	8	0	0	28

Pwt means parent individuals with wild-type character, Pm indicates parent individuals with mutant phenotype, F_2_m indicates number of individuals with mutant phenotype in F_2_ population, F_2_wt indicates individual number of F_2_ population with wild-type character.

**Fig 3:**
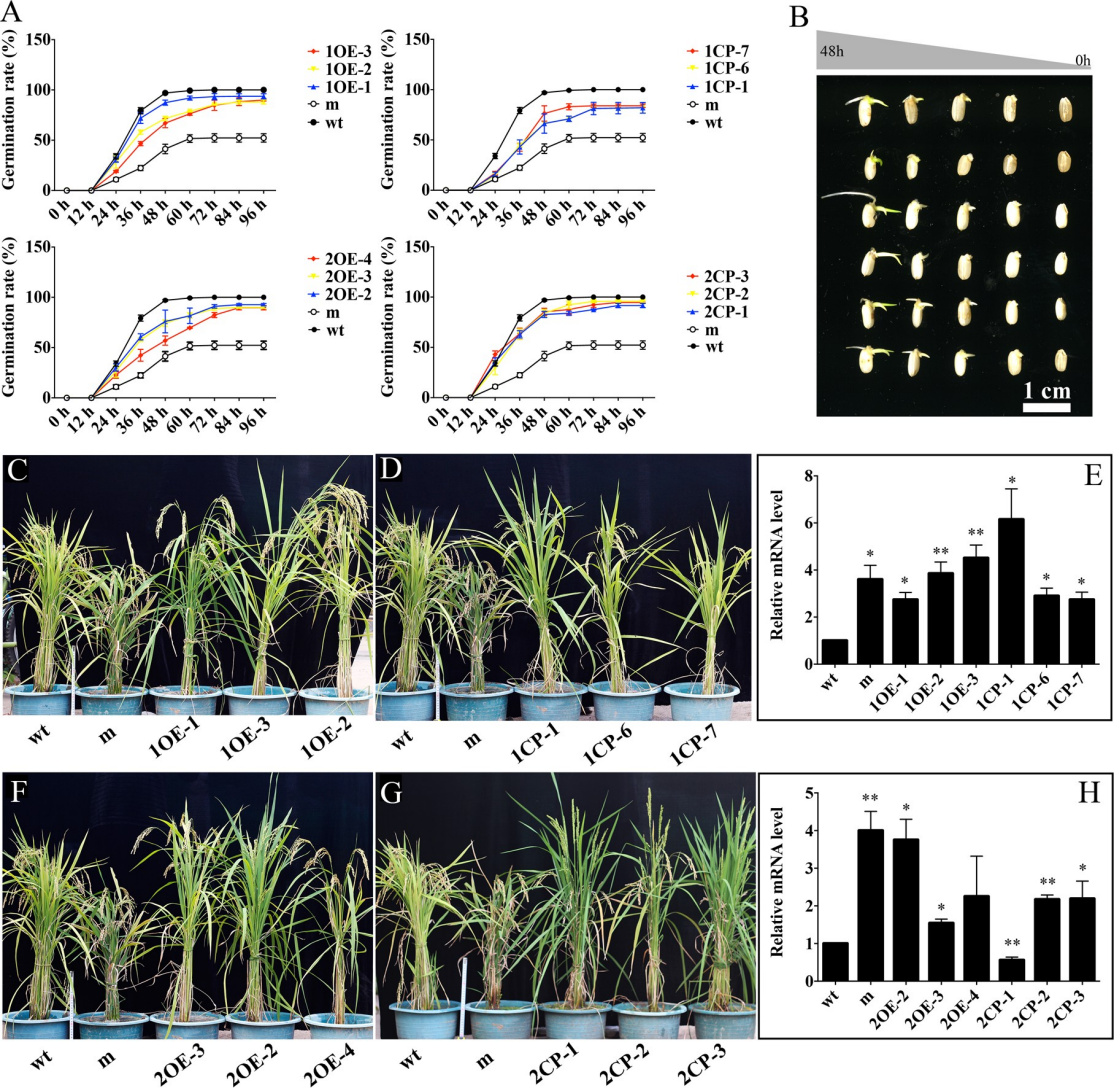
Genetic complementation analyses of the mutant. (A) Germination assay of wild type, mutant, and different transgenic lines. Upside and downside panels are the data for the overexpression (left) and native promoter lines (right) of *OsKO1* and *OsKO2*, respectively. (B) Images showing the germinating seeds from 0 to 48 h of different rice lines. (C) Rescue of the mutant phenotype in *OsKO1*overexpression lines. (D) Rescue of the mutant phenotype in *OsKO1* transgenic lines driven by its native promoter. (E) Expression of *OsKO1* gene in different rice lines. (F) Rescue of the mutant phenotype in *OsKO2* overexpression lines. (G) Rescue of the mutant phenotype in *OsKO2* transgenic lines driven by *OsKO1* native promoter. (H) Expression of *OsKO2* gene in different rice lines. “wt”, wild-type; m, mutant; 1OE-1,2,3, *OsKO1* gene overexpression transgenic lines; 1CP-1,6,7, *OsKO1* gene native promoter transgenic lines; 2OE-2,3,4, *OsKO2* gene overexpression transgenic lines; 2CP-1,2,3, *OsKO2* gene native promoter transgenic lines. Rice *OsActin1* was used as internal reference for the qRT-PCR analysis. Data are means±SE (n≥3). Significant difference was determined by Student’s t-test (*p < 0.05, **p < 0.01).

To confirm the mutant phenotype is the resultant of the mutations on *OsKO1* gene, we conducted genetic complementation analyses in the mutant background. For complementary experiment, two types of transgenic plants were generated. The first one was generated with the vector containing a maize ubiquitin promoter and full length of *OsKO1* cDNA from wild-type. The second one was conducted through the vector containing the native promoter (~2 kb) and full ORFs sequence of the gene. More than ten positive transgenic plants were obtained for each type of transformation, of which all rescued the mutant phenotype (**Fig 3A–3D**). The expressions of *OsKO1* in all the transgenic lines were enhanced (**Fig 3E**). Interestingly, the expression of *OsKO1* in the mutant is also higher in mutant than that in wild type (**Fig 3E**). Altogether, mutations on *OsKO1* gene resulted in the mutant phenotype.

Since OsKOs are P450 family enzymes, the C-terminal region of this family is the heme-binding domain, which is quite conserved [34]. To see the conservative of the mutated site, we than conducted alignment analysis with the sequences from the five OsKO proteins and KO proteins involving in GA biosynthesis from Arabidopsis, wheat and *Nelumbo nucifera*. The result showed that sequences of these eight KO proteins are highly conserved with more than 70% similarity (**S2 Fig**). Previous study has shown that *OsKO1*, *OsKO2* and *OsKO5* were associated with the dwarfism phenotype, and have closer relationship to each other than with *OsKO3* and *OsKO4* [29]. Although the mutated site is not highly conserved, it prefers to be an–OH containing amino acid residue (**S2 Fig**). The replacement of T by M might lead to minor structural changes in the center of enzyme (**S1B Fig**). Based on these, full-length cDNA sequence of *OsKO2* was also applied to conduct the complementation analysis under the same systems as *OsKO1*. It could also rescue the mutant phenotype. Interestingly, although its rescuing on the germination is weaker than *OsKO1* (**Fig 3A and 3B**), it has a stronger rescuing on the semi-dwarfism phenotype, especially under the native promoter driving (**Fig 3F and 3G**). The expression of *OsKO2* is also higher in the mutant, but with irregularly changes in different transgenic lines (**Fig 3H**).

### OsKO1 contains ent-Kaurene oxidase enzyme activity and involved in GA biosynthesis in rice

**Fig 4:**
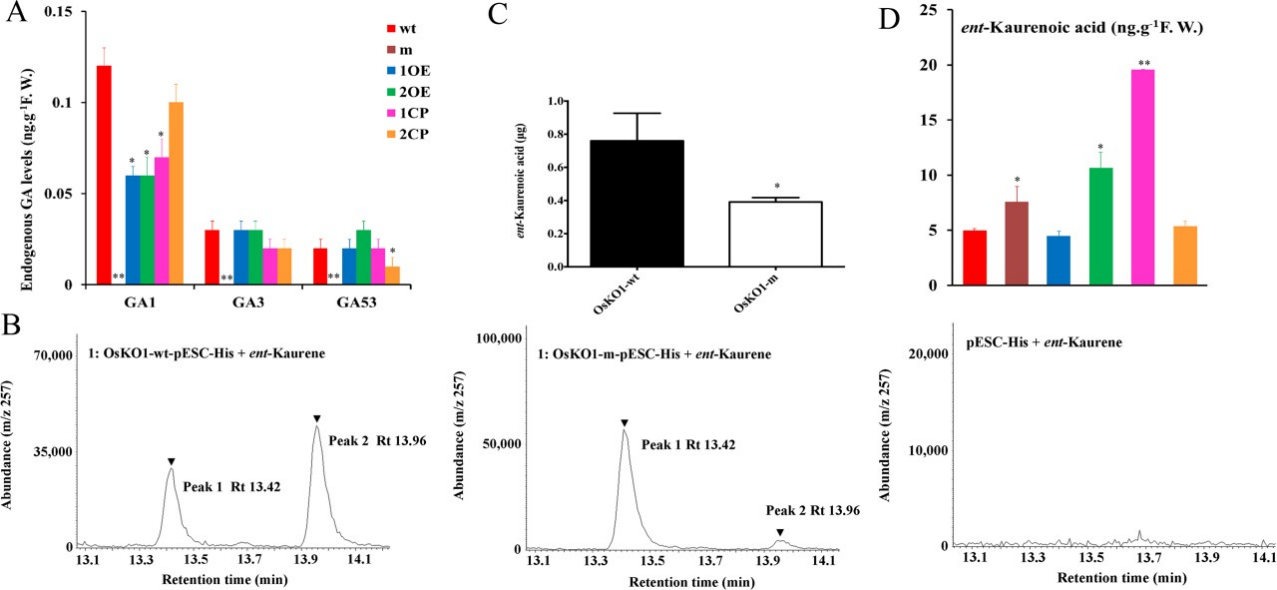
GA contents and OsKO1 enzyme activity analyses. (A) Endogenous GA_1_, GA_3_ and GA_53_ contents in the embryos of different rice lines. (B) Yeast expression and GC-MS analysis of enzyme activity of normal OsKO1 (left panel) and mutated OsKO1 (middle panel), with empty vector pESC-His transgenic yeast as negative control (right panel). GC-MS chromatograms at single ion 257 showed products *ent*-kaurenol (peak 1) and *ent*-kaurenoic acid (peak 2) catalyzed by OsKO1 from *ent*-kaurene. The right panel. (C) Quantitative analysis on the production of *ent*-kaurenoic acid between normal OsKO1 and mutated OsKO1 in the transgenic yeast system. Data are means±SE (n = 3). (D) Endogenous content of *ent*-kaurenoic acid in one-month-old seedlings of different rice lines. Wild-type (wt), mutant (m) and transgenic lines in mutant background. 1OE, *OsKO1* gene overexpression lines; 2OE, OsKO2 overexpression lines; 1CP, *OsKO1* gene native promoter transgenic lines; 2CP, *OsKO2* gene native promoter transgenic lines. Significant difference was determined by Student’s t-test (*p < 0.05, **p < 0.01).

Since the exogenous GA could rescue the mutant phenotype, we analyzed the contents of GA_1_, GA_3_ and their precursor GA_53_ in the wild-type and mutant. All of them were almost undetectable in the mutant, much less than that in the wild-type (**Fig 4A**). Consistent with the genetic complementation analyses, the GA contents could also be recovered in the transgenic mutant lines of both *OsKO1* and *OsKO2* genes (**Fig 4A**). Previous studies proved the *ent*-kaurene oxidase activity of OsKO2, which catalyzes the reaction from *ent*-kaurene to *ent*-kaurenol then to *ent*-kaurenoic acid in GA biosynthetic pathway [27]. Combined all these data, we hypothesize that OsKO1 might also catalyze the same reaction, and the mutation might attenuate its enzyme activity. To verify this hypothesis, transgenic yeast strains expressing wild-type and mutated *OsKO1* on pESC-His vector were generated and used to conduct the enzyme assay as described in M&M. In the wild-type system, both *ent*-kaurenol and *ent*-kaurenoic acid could be produced, with more *ent*-kaurenoic acid detected (**Fig 4B, S3 Fig**). However, much less *ent*-kaurenoic acid was produced in the mutant system (**Fig 4B and 4C**). We then analyzed the endogenous content of *ent*-kaurenoic acid in rice tissues. Although there were undetectable *ent*-kaurenoic acid in the germinating seeds, it is detectable in the one-month-old seedling. Surprisingly, the content of *ent*-kaurenoic acid in the mutant is a little bit higher than that in the wild type (**Fig 4D**). Together, these results indicated that OsKO1 contains the enzyme activity to catalyze the producing of *ent*-kaurenol and *ent*-kaurenoic acid in GA biosynthetic pathway. The mutation from Thr to Met did not affect its activity to catalyze the reaction from *ent*-kaurene to *ent*-kaurenol, but decreased its activity to catalyze the *ent*-kaurenol to *ent*-kaurenoic acid.

### Subcellular localization and expressional pattern of OsKO1

**Fig 5:**
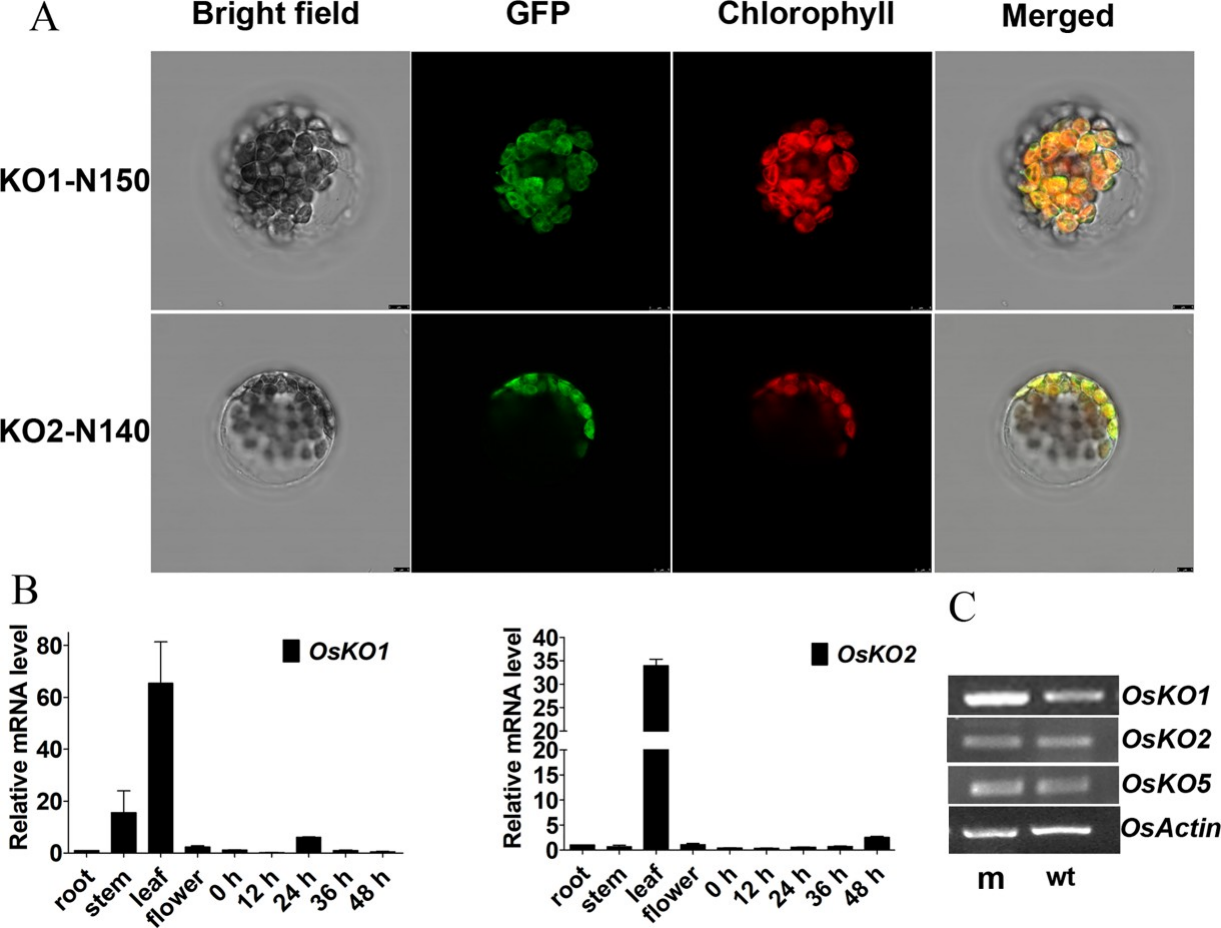
Subcellular localization of OsKO1 and OsKO2, and expression analyses on their encoding genes. (A) Subcellular localization of OsKO1 and OsKO2. OsKO1-N150 and OsKO2-N140 indicates the N-terminal 150 and 140 amino acids of OsKO1 and OsKO2, respectively. The N-terminal regions containing transit peptides of OsKO1 and OsKO2 were fused to GFP, and transiently expressed in *Arabidopsis* mesophyll protoplasts, respectively. Scale bars = 2.5 μm. (B) Expressional patterns of *OsKO1* and *OsKO2*. Rice *OsActin1* was used as internal reference for the qRT-PCR analysis. Data are means±SE (n = 3). The 0, 12, 24, 36 and 48 h stand for the embryos from the rice seeds at different time after imbibition, respectively. (C) Comparison of the expressional levels of *OsKO1*, *OsKO2* and *OsKO5* in the rice seed at 24 after imbibition between mutant and wild-type.

Previous study in *Arabidopsis* has shown that the AtKO1 protein localizes on the out envelope membrane of plastids [22]. To determine the subcellular localization of OsKO1, we applied transient transfection assay in *Arabidopsis* protoplast in parallel with OsKO2. When the full length CDS of *OsKO1* and *OsKO2* genes were fused with *GFP* respectively, no GFP signal could be detected in both constructs, although there was strong signal in the cell for the empty pGFP2 vector control (**S4 Fig**). Previous study has shown that transit peptides at the N-termini for OsCPS1 and OsCPS2 are enough for their transport into plastid [35], we then used the N-terminal 150 amino acids of OsKO1 and N-terminal 140 of OsKO2 proteins containing transit peptides to fuse with GFP, respectively. The signal of OsKO1 could be detected at both plastid and other membrane system, whereas, OsKO2 signal could be only detected at plastid membrane (**Fig 5A**). This is generally consistent with the result in *Arabidopsis* [22]. The expressional patterns of *OsKO1* and *OsKO2* were also analyzed through real-time PCR. Although there are tiny difference, both of them are ubiquitous expressed among different tissues detected in this study with the highest level in leaf (**Fig 5B**). The expression of *OsKO1* was higher than that of *OsKO2* in the germinating embryo and stem from young seedlings (**Fig 5B**). Since OsKO1, OsKO2 and OsKO5 were all shown to be involved in GA biosynthesis, we checked the expression of these three genes in the germinating seeds of both wild-type and mutant at 24 h after imbibition. It is shown that all of them expressed higher in mutant than wild-type, with *OsKO1* having the highest expressional level (**Fig 5C**).

### Pathways affected by the mutation that lead to the delayed germination

To further explore the pathways through which *OsKO1* regulate seed germination in rice, RNA-seq was used to profile the differentially expressed genes (DEGs) in seeds at 36 h after imbibition among wild-type, mutant, and mutant treated with 10^−5^ M GA (**S2 Table, S3 Table**). For selecting DEGs, the threshold was set at *p* < 0.05. Totally, 281 genes were differentially expressed between wild-type and mutant, including 174 up-regulated and 107 down-regulated genes in wild-type compared to mutant (**S4A Fig**); 497 differentially expressed genes were detected between mutant treated with exogenous GA and mutant, including 259 up-regulated and 238 down-regulated genes in mutant treated with exogenous GA compared to mutant (**S5A Fig**). The reliability of eight freely selected DGEs were further confirmed by qRT-PCR (**S5B Fig**). Unsurprisingly, the ABA-responsive genes, including those encoding seed storage proteins, seed maturation proteins, and LEAs proteins, had higher expression in mutant compared to both wild-type and mutant treated with GA. To the contrary, genes encoding MYB type transcription factors, sugar and lipid transporters, hydrolase and cytochrome P450 family proteins were down-regulated in mutant (**S3 Table**). As we have reported, regulation at translational level is more important than that at transcriptional level for rice seed germination [36]. We then compared the proteome profiles of the germinating embryos between wild-type and mutant through a gel-based proteomic strategy. Among the 33 significantly changed proteins, 8 up-regulated proteins in mutant were seed storage proteins (U50, T44, T46) and ABA responsive late embryogenesis abundant proteins (U2, U32, U47, U49, U51) (**S5C Fig, S3 Table**). We then compared the DEGs list and the changed protein list. Although the number of overlapped genes was very limited, the ABA responsive LEAs and seed maturation proteins existed in both lists, and showed higher expression at both mRNA and proteins levels in mutant than wild-type (**S3 Table, S4 Table**).

**Fig 6:**
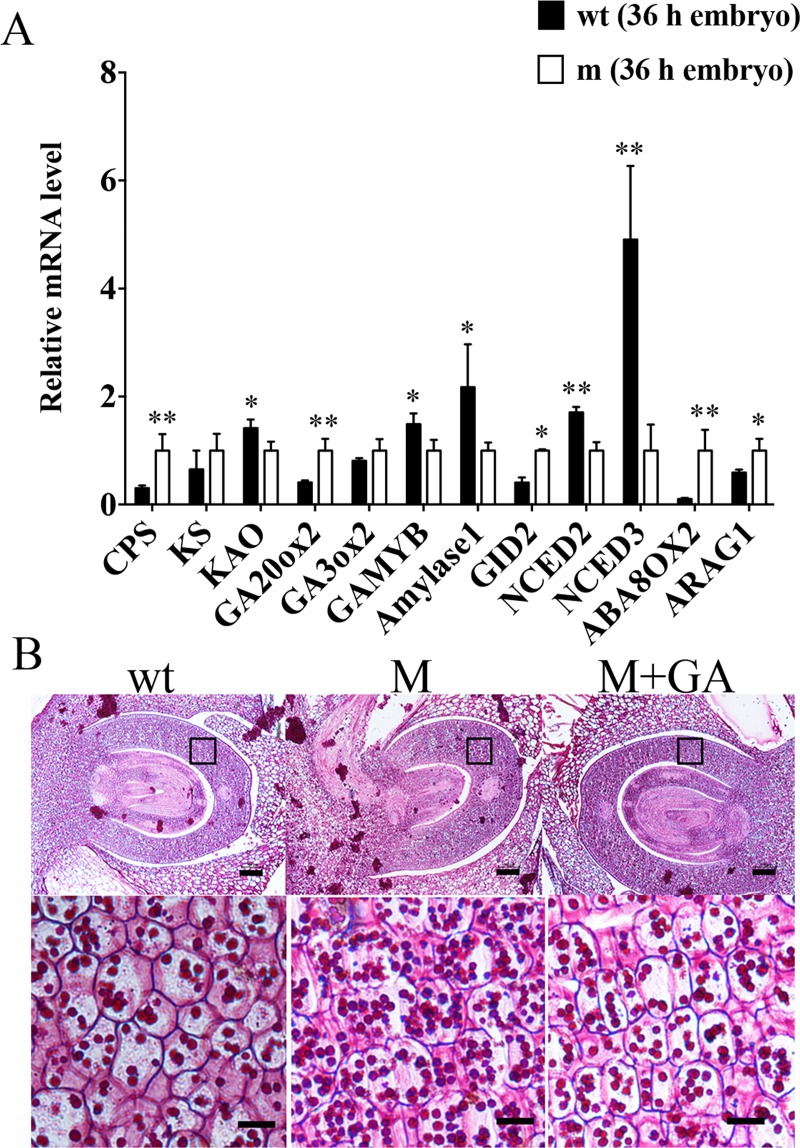
Analyses on the expression of GA and ABA metabolism and responsive genes and the degradation of starch. (A) Comparison on the expression of GA and ABA metabolism and responsive genes between the embryos from wild-type and mutant through qRT-PCR. Data are means±SE (n = 3). Significant difference was determined by Student’s t-test (*p < 0.05, **p < 0.01). (B) Microscope observation of the starch granules in the paraffin sections of rice embryos from wt, m and m treated with exogenous GA_3_ (m+GA). The down-side panels are the enlaged rectangle areas of their corresponding up-side panels. Bars = 15μm. All the embryos were from the seeds at 36 h after imbibition.

To further confirm the involvement of GA and ABA signaling, we then compared the expression of GA- and ABA-responsive and metabolic genes in the germinating embryos between wild-type and mutant through RT-PCR. Although most of the GA biosynthetic genes were up-regulated, the GA-responsive genes, such as *α-amylase* and *GAMYB*, were down-regulated in mutant related to wild-type (Fig 6A), which was similar with that in *gd1* and *dsg1* mutants [8,15]. *GID1*, a positive regulator of GA signaling, was also up-regulated in mutant (Fig 6A). Interestingly, ABA biosynthetic genes, *OsNCED2 and OsNCED3*, were down-regulated, and ABA catabolic gene (*OsABA8ox2*), playing key roles in ABA catabolism [37–40], was up-regulated, although ABA-responsive gene *ARAG1* was up-regulated in the mutant (**Fig 6A**). Considering that the GA induced expression of *α-amylase* is very important for the degradation of starch in cereal seed, we then detected starch granule in the germinating rice seed through microscopy. Obviously, there were more starch granules in the mutant than wild-type and mutant treated with exogenous GA (**Fig 6A**). Altogether, it seems the deficiency of GA biosynthesis lead to the delay of ABA signaling attenuation and starch degradation, which might result in the delayed radicle protrusion phenotype in the mutant.

## Discussion

### Analysis of the germination delayed dwarfism rice mutant

Seed germination is an important agronomic trait for rice production. Seeds fail to germinate or germinate on mature rice plant (pre-harvest sprouting) will both decrease its yield. As has been reported in *Arabidopsis* [41–43], seed germination is mainly controlled by the homostasis of plant hormones GA and ABA, which has not been fully verified in rice. Recently rice seed germination mutants were characterized based on either T-DNA insertion mutant cloning or map-based cloning methods [8,15,20]. In this study, we identified an mutant showing delayed seed germination and semi-dwarfism phenotype through screening the EMS induced rice mutants. Using whole-genome re-sequencing on F_2_ progeny bulks, two continuous mutations on *OsKO1* gene were detected to be responsible for the delayed germination and semi-dwarfism of mutant. The *osko2* mutant was previously reported as *d35* mutant [26], with single mutation on the fifth exon leading to semi-dwarfism and an insertion in the 4^th^ exon leading to serious dwarfism or even death [26]. However, involvement of *OsKO2* in seed germination was from an indirect study of *OsLOL1* [44]. In our study, the two continuous mutations on the eighth exons of *OsKO1* lead to a transition from Thr to Met, which results in the delayed seed germination and semi-dwarfism phenotype. The complementary experiments showed that either *OsKO1* or *OsKO2* gene could rescue the mutant phenotype (**Fig 3**). Complementary expression of each of these two genes could recover the endogenous GA content as well (**Fig 4**). In addition, exogenous GA could also rescue the mutant phenotype. Together, it could be concluded that *OsKO1* gene could affect seed germination through their function in GA biosynthesis.

### Function verification of *OsKO1* gene

There are five similar *KO* genes in rice genome. Phylogenic analysis sorted them into two clades, with *OsKO1*, *OsKO2* and *OsKO5* genes belonging to clade I, and *OsKO3* and *OsKO4* belonging to clade II [26,29]. Among them, *OsKO3* and *OsKO4* might involve in phytoalexin biosynthesis, and hence contribute to disease resistance [26,29,45]. *OsKO2* has been proven to be involved in GA biosynthesis [26]. Studies showed that *OsKO1* and *OsKO5* might be also involved in GA biosynthesis [26,29,46]. Chen *et al*. [29] proposed that OsKO1, OsKO2 and OsKO5 might form into heterotrimer to catalyze the reaction in GA biosynthesis. However, previous report [27] and the yeast system enzyme assay in this study showed that both OsKO1 and OsKO2 could independently catalyze the reaction. Furthermore, there is only one KO gene in Arabidopsis genome, it is impossible to function in heterotrimer. Expression analyses of *OsKO1* and *OsKO2* from different tissues, including root, stem, leaf, flower, embryo at 0, 12, 24, 36, and 48 h after imbibition, showed that *OsKO1* is highly expressed at the early stage of seed germination and in the stem of young seedling, which is different with that of *OsKO2* (**Fig 5**). Except for the delayed germination, phenotyping also showed that the semi-dwarfism is mainly ascribed to the shortening of the first and second internodes (**Fig 1B**). Combining with the fact with *OsKO2* rescuing weakly on the germination, it seems that *OsKO1* is the major one that functions in GA biosynthesis during seed germination and young seedling stage in rice.

In GA biosynthetic pathway, KO catalyzes the reaction from *ent*-Kaurene to *ent*-Kaurenoic acid via *ent*-Kaurenol and *ent*-Kaurenal [23,24]. The enzyme activity of OsKO2 has been confirmed in previous study [27]. Consistent with the fact that *OsKO1* could rescue the mutant phenotype (**Fig 3**), it could also catalyze the reaction from *ent*-Kaurene to *ent*-Kaurenoic acid *in-vitro* (Fig 4B and 4C). Since GA content in the *OsKO1* complementary transgenic rice was recovered as well (**Fig 4A**), it could be concluded that OsKO1 also contains the same enzyme activity as OsKO2 and involves in GA biosynthesis. However, the endogenous *ent*-Kaurenoic acid in mutant is higher than that in the wild type in the one-month-old seedling. It seems that there is compensation in the mutant by OsKO2 after the early seedling growth stage. The mutation of Thr to Met in this mutant resulted in the decrease of its catalyzing efficiency to produce *ent*-Kaurenoic acid, with no effect on the producing of *ent*-Kaurenol (**Fig 4B**). Sequence alignment showed that the amino acids around the mutated site were highly conserved with the mutated site preferring to be Thr (**S2 Fig**). It seems that each of the reactions from *ent*-Kaurene to *ent*-Kaurenol then to *ent*-Kaurenal and finally to *ent*-Kaurenoic acid is catalyzed by different motif of KO. Based on the current study, the last conserved motif might not involve in catalyzing the producing of *ent*-Kaurenol, but in the producing of either *ent*-Kaurenal or *ent*-Kaurenoic acid. Further studies are still needed to determine the exact functional motif for each reaction. Taken together, *OsKO1* is another functional *OsKO* gene on early GA biosynthesis pathway especially in *ent*-kaurenol into *ent*-kaurenoic acid in rice.

### GA and ABA on the control of rice seed germination

GA and ABA are two antagonistic phytohormones, the defect in GA biosynthesis will enhance ABA signaling [47]. It is believed that the balance between GA and ABA rather than their absolute contents controls the seed germination process [4]. LEAs are typical ABA-responsive proteins and involved in many abiotic stress response [48,49]. In rice seed, LEAs along with seed maturation and seed storage proteins are accumulated in responding to the enhancing ABA signal during seed maturation, which will help the seed to survive from desiccation, and maintain its longevity [50,51]. At the early stage of seed germination, these proteins will be degraded to provide precursors and energy for seedling establishment [52]. RNA-seq data showed that the transcripts of these genes were much higher in mutant than wild-type, suggesting the enhanced ABA signaling, which is also supported by the higher level of *ARAG1* transcript (Fig 6A) in the mutant. In addition, the degradation of LEAs, seed maturation and seed storage proteins were much slower than that in wild-type (**S5C Fig, S4 Table**), indicating the weakening of GA signaling, since the mobilization of reserves is promoted by GA. In cereal seed germination, starch degradation catalyzing by α-amylase is a typical activity [16,53], which is obviously weakened in the mutant (**Fig 6B**). This weakening could be ascribed to the down-regulation on the expression of *α-amylase* at both mRNA and protein levels (**S3 Table, S4 Table**), which might also be the result of up-regulation of *ARAG1* [54]. Meanwhile, *GAMYB* is also down-regulated in mutant relative to wild type (**Fig 6A, S3 Table**). Together, it could be concluded that mutations on OsKO1 and OsKO2 lead to defect in GA biosynthesis, which results in the weakening of GA signaling and enhancing of ABA signaling, and hence delay the germination process. To the contrast of GA and ABA signaling results, most of the GA biosynthesis genes, ABA catabolic gene and *GID2*, a positive GA regulator [55–57] were up-regulated, whereas, ABA biosynthesis genes were down-regulated in the mutant (Fig 6A). It seems there is feedback regulation to re-balance the GA and ABA in the mutant during germination.

## Materials & methods

### Plant materials, germination condition and GA treatment

The rice germination delayed and dwarfism mutant (hereafter named as mutant) was derived from EMS mutagenesis of *Oryza sativa* L. cv. Nipponbare (hereafter named as wild-type, and abbreviated as wt) and planted in paddy field in Wuhan, China. For measuring germination rates under GA treatment, 50 seeds of wt and mutant harvested at late September from three biological replicates were germinated on wet filter paper treated with sterilized water or 10^−5^ M GA (SIGMA, USA) at 25 ^o^C constant temperature incubator with 12 h light (3 Lux)/ 12 h dark cycle, 70% relative humidity. To test whether the mutation is on GA biosynthesis pathway and GA responsive, 20 seeds of wt and mutant were surface sterilized with 3% NaClO for 30 min, then washed with sterilized water for three times. After sterilization, wt and mutant seeds were separately placed on 1% agar treated with 10^−12^–10^−4^ M GA. Length of the second leaf sheath was measured after one week as previous described [28].

### Whole-genome resequencing of F_2_ progeny bulks DNA

F_2_ progenies derived from crossing then self-crossing of mutant with its parental line Nipponbare were bulk sequenced. In details, 60 individuals with mutant phenotype and 60 individuals with wild-type character from the F2 population were sequenced as previously described [58]. A total of 23.13 Gb parental line sequence reads and 24.28 Gb reads of mutant from Illumine Hiseq 2000 PE100 sequencer were controlled by fastqc software to get high quality reads. Clean reads were aligned to reference genome Os-Nipponbare-Reference-IRGSP-1.0 from the Rice Annotation Project by bwa 0.5.9-r16 [59,60]. Then the bam file after alignment was sorted and removed duplications by software samtools 0. 1. 18 [61]. After removing duplications, the file was realigned to reference genome to reduce false positive through GATK Indel Realigner. GATK Unified Genotyper was used to find SNPs and indels, then the SNPs and InDels were filtered through GATK VariantFiltration software to get 200508 filtered SNPs between mutant and Nipponbare [62]. SNP index was calculated according to previous study [63]. For checking linkage of mutations with mutant phenotype, taqman probe 5’-FAM-TGCAGGCAATGCACATCG-BHQ-1-3’ and primer pair 5’-ATGAACAGGAAGGAGTGGGAGT-3’, 5’-TGGAGCTTGTAGGCGGTGAG-3’ (**S5 Table**) were used. Functional concentration of probe is 1 pmol and was detected by CFX96 Real Time System (BIO-RAD, USA) at 95°C 10 min, 92°C 15 sec, 60°C 1 min, 40 cycles according to previous methods with modifications [64,65].

### Sequence analysis

CDS sequence of mutated gene was cloned and aligned to sequence from RAP-DB (http://rapdb.dna.affrc.go.jp). Conserved domain annotation of cytochrome P450 genes were referenced from EMBL-EBI (http://www.ebi.ac.uk/interpro/search/sequence/).

### Function identification of OsKO1 through yeast expression system and GC-MS analysis

The ORF of *OsKO*1 gene from wild-type rice and mutant rice were separately cloned into pESC-His vector under the restriction sites EcoR I and Spe I. The two plasmids OsKO1-wt-pESC-His, OsKO1-m-pESC-His were transformed into WAT11 yeast strain that express the *Arabidopsis thaliana* reductase NADPH-CYP under the control of a Gal1 promoter using previously reported lithium acetate method. After incubating the single colony of transgenic strain in synthetic dropout medium that lack His amino acid contained 2% glucose to OD 0.8, the expression of *OsKO* genes were induced by diluted the transgenic strain cultures in the same SD medium contained 2% galactose. The substrate *ent*-kaurene was added to a final concentration of 100 μM and co-incubated with *OsKO* or pESC-His empty vector transgenic strains for 72 hours. After extracted with ethyl acetate, the product was dried with N_2_ gas and then trimethylsilylated with N-methyl-N-trimethylsilyltrifluoroacetamide (MSTFA) and analyzed by GC-MS. Three independent biological replicates with different colonies were conducted for the whole procedure. For quantification of the first product *ent*-kaurenol, 0.1 μg, 0.5 μg, 1 μg, 5 μg, 10 μg *ent*-kaurenol standards and their relative GC-MS area were used to depict standard curve, then to calculate product content. To quantify another product *ent*-kaurenoic acid, 0.1 μg, 0.5 μg, 1 μg, 5 μg, 10 μg *ent*-kaurenoic acid standards were used and standard curve were depict based on the same method with *ent*-kaurenol.

GC-MS analysis was carried out on 7890 A gas chromatograph with triple-axis detector coupled with 5975 C mass spectrometer (Agilent, USA). Parameters of mass spectrometer and gas chromatograph were set as: ionization voltage, 70 eV; ion source temperature, 80°C; solvent delay, 4 min; mass to charge ratio range, 40 to 400 amu; flow rate of helium carrier gas, 1 mL/min. Samples were injected into a HP-5 MS column (30 m × 0.25 mm × 0.25 μm film thickness, Agilent) in the splitless mode with outlet temperature 250°C. The column temperature was programmed as: hold at 80°C for 1 min, and a linear gradient of 30°C min^-1^ to 200°C, from 200°C to 280°C at 5°C min^-1^ hold at 280°C for 2 min.

### Construction of complementation, overexpression vectors for rice transformation

The full length of *OsKO*1 and *OsKO*2 CDS under the control of 2075 bp native promoter with 5’ UTR was inserted into binary vector pCAMBIA 1301 by HindIII, KpnI digestion and then transformed into mutant according to previous method with modification [26] to conduct the complementary experiment. For overexpression of *OsKO1* and *OsKO*2 in mutant, cDNAs were cloned into modified pCAMBIA 1301 under the control of maize ubiquitin promoter. Plant transformation was with Agrobacterium strain EHA105 and according to previous study [66]. After using MS medium contained 30 μg/mL hygromycin to select homozygous transgenic plants from T1 generation, phenotypes on the transgenic plants were observed.

### Analysis of gibberellins and *ent*-kaurenoic acid contents

Nipponbare wild-type and mutant seeds freshly harvested at the same time were surface sterilized with 3% NaClO, then germinated on MS medium at 25°C, 70% humidity with 12 h light (3 Lux)/ 12 h dark cycle constant incubator for 36 h. A total of 1 g embryos of wild-type and mutant seeds were excised and snap frozen in liquid nitrogen for GA measurement. Endogenous GA contents of 3 g leaves of *OsKO1* overexpression and complementary transgenic lines grow for 64 d selected by 30 μg/mL hygromycin were compared to Nipponbare and mutant. Endogenous GA quantification method was according to previous study [67].

For *ent*-kaurenoic acid measurement, 100 mg tissues were ground into fine powder in liquid nitrogen, and extracted with methanol containing 1% FA through vortexing 2 min and 12 h at 4°C in darkness. After centrifugation at 12000 *g* for 10 min, the supernatant was harvested, and subjected to C18 matrix, and then dried. Resuspended it with methanol containing 1% FA, and centrifuged again. The supernatant was harvested and subjected to UHPLC (Thermo Scientific Ultimate 3000) and MS (Thermo Scientific TSQ Quantiva) analysis.

### RNA isolation, quantitative real time PCR and digital gene expression profile

Total RNAs of root, stem, leaf, flower, and embryos at 0, 12, 24, 36 and 48 h after germination were extracted with TRIzol Reagent (Invitrogen, Carlsbad, USA) to check the expression profiles of *OsKO1* and *OsKO2* genes. For qRT-PCR and differentially expressed genes (DEGs) analysis with digital gene expression (DGE) profile, embryos of wild-type, mutant and mutant with 10^−5^ M GA germinated for 36 hours were used [68,69]. Three independent biological replicates were performed for qRT-PCR and DGE assay, respectively. For qRT-PCR, cDNAs were obtained through Reverse Tra Ace-α-First Strand cDNA synthesis Kit (TOYOBO, Osaka, JAPAN) with 2 μg of total RNA after DNase I digestion. qRT-PCR was performed on CFX96 Real Time System (BIO-RAD, USA) with SYBR green fluorescence according to manufacturer’s instructions. OsACTIN1 was used to normalize the expression ratio of each gene. For DGE sequencing (Illumina Hiseq platform), a total amount of 3 μg RNA was used for each library construction. Reads were mapped to the reference genome with software TopHat v2.0.12. For quantification of gene expression level and differentially expressed genes analysis HTSeq v0.6.1 and DESeq 1.10.1 were used. Quantification of genes used expected fragments per kilobase of transcript per million fragments sequenced (FPKM) based on gene length and reads count mapped to the gene [70]. Genes with p value < 0.05 found in DESeq analysis were assigned as differentially expressed.

### Paraffin section and starch granule staining

Leaf sheath and internode of wt and mutant were fixed in FAA solution contained 5% glacial acetic acid, 5% formaldehyde, 70% ethanol until use. The samples were then undergone dehydration, infiltration and embedded in paraffin. After cutting into sections, they were preserved by neutral balsam. For starch granule staining, embryos of wt, mutant and mutant with GA treatment seeds germinated for 36 h were fixed and then embedded in paraffin. Section cutting method, Periodic Acid Schiff staining and specimen observation was according to previous study [36,71]. At least three replicates were fixed and observed for each sample.

### Two-dimensional electrophoresis (2-DE)

Four independent biological replicates of mutant and wild-type rice seeds at 36 h after imbibition were sliced to get embryos. After extracting by acetone precipitation method, 1 mg proteins were separated by 17 cm, PH 4–7 IEF gels (BIO-RAD, USA) with isoelectric focusing and SDS-PAGE. Four independent replicate gels were run and analyzed in parallel for each sample. PDQuest 2-DE Analysis Software (BIO-RAD) was used to analyze and select proteins with significant changes (folds change>2, *p*<0.05). Changed protein spots were digested and desalted, and then peptide solution was identified by a MALDI-TOF mass spectrometer voyager STR (AB, Milwaukee, WI, USA) and searched against NCBI rice database (contains 63553 sequences and 22360987 residues) with Mascot software (http://www.matrixscience.com). Search parameters were set as: type of search, peptide mass fingerprint; enzyme, trypsin; fixed modifications, carbamidomethyl (C); protein mass, unrestricted; mass values, monoisotopic; peptide mass tolerance, ±200 ppm; peptide charge state, 1+; max missed cleavages, 1. Protein score is -10*Log(P), protein scores greater than 61 are significant (p < 0.05). Protein with confidence level > 95% were credible. Protein extraction, 2-DE, in gel digestion and desalting methods were all according to previous study [72].

### Subcellular localization assay

ORFs encoding full length of OsKO1 and OsKO2 with its stop codon changed into Arg were fused to GFP under the control of CaMV 35S promoter into pGFP2 vector through XhoI, KpnI sites for OsKO1. The cDNA fragments encoding N-terminal 150 and 140 amino acids of OsKO1 and OsKO2 was cloned into pGFP2 vector through the same restriction endonuclease recognition sites with vectors for ORFs, respectively. Each constructed plasmid was introduced into four-week old mesophyll protoplasts of *Arabidopsis* wild-type (Col-0) through PEG-calcium mediated transformation to transiently express OsKO-GFP fusion proteins according to reliable guidelines [73]. GFP signal was detected by Leica TCS SP8 laser scanning confocal microscope (Leica, Germany) with same exciting light wavelength 488 nm, detection light wavelength 500 to 530 nm for GFP and 600 to 630 nm for chloroplast.

## Supporting information

S1 Table Genes that exist in the region associated with mutant phenotype. (PDF)

S2 Table Summary of RNA-seq data generated by Illumina Hiseq platform. (PDF)

S3 Table Significantly differentially expressed genes. (PDF)

S4 Table MALDI TOF/TOF MS identified significantly changed proteins. (XLSX)

S5 Table Primers used in this study. (PDF)

S1 Fig Distribution and sequence alignment analyses of *OsKO* genes. (A) Contiguously arranged five *OsKO* genes in a 120 kb highly linked region on the sixth chromosome of rice genome. The row marked by red filled circle shows the old nomenclature of *OsKO genes*, and that marked by green filled circle shows the updated name of each *OsKO gene*. (B) Three-dimensional structures of the wild type (left panel) and mutated (right panel) OsKO1 proteins. The green arrow indicates the mutated site.
(PDF)

S2 Fig Sequence alignment analysis of KO proteins from rice, Arabidopsis, wheat and Nelumbo nucifera. The read rectangle and arrow indicate the mutated site.
(PDF)

S3 Fig (A) Chromatograph and mass spectra of ent-kaurene, ent-kaurenol, and ent-kaurenoic acid standards. The three panels at left side are the chromatograph images, and those at the right side are the corresponding mass spectra for each chemical. (B) GC-MS analysis on the amounts of intermediate product *ent*-kaurenol (peak 1) and final product *ent*-kaurenoic acid (peak 2) catalyzed by wild type (left panels) and mutated OsKO1 (right panels) from *ent*-kaurene at 10 (up panels) and 20 min (bottom panels).
(PDF)

S4 Fig Subcellular localization analysis of OsKO1 and OsKO2 with their full length CDS fused with GFP protein. The GFP containing vector was used as control. Scale bars = 2.5 μm.
(PDF)

S5 Fig RNA-seq and 2-DE analyses on the mutant and wild type rice. (A) Volcano plots showing the differentially expressed genes between wt and m (upside image), and m and m+GA (bottom image). mGA stands for m+GA. **(B)** Verification of the RNA-seq results through qRT-PCR on the selected genes in GA biosynthesis and ABA signaling pathways. (C) 2-D gel images of wt and m embryos. U and D indicate the up- and down-regulated proteins in mutant, respectively.
(PDF)
