# Supplementary material for: Mutations on ent-kaurene oxidase 1 encoding gene attenuate its enzyme activity of catalyzing the reaction from ent-kaurene to ent-kaurenoic acid and lead to delayed germination in rice
Source: PLoS Genet. 2020 Jan 10;16(1):e1008562. doi: 10.1371/journal.pgen.1008562 (PMC6977763; doi:10.1371/journal.pgen.1008562)
Supplement: S1 Table — (PDF) [file pgen.1008562.s001.pdf]

Table S1. Genes that exist in the region associated with mutant phenotype.

Gene ID	Annotation
Os06t0569601-01	Hypothetical gene.
Os06t0569900-01	Similar to Ent-kaurene oxidase 1
Os06t0570100-01	Ent-kaurene oxidase 2.
Os06t0570600-00	Similar to Ent-kaurene oxidase 5.
Os06t0570900-00	Helix-loop-helix DNA-binding domain containing protein
Os06t0571300-01	Similar to predicted protein.
Os06t0582600-01	Similar to Cysteine proteinase.
Os06t0595900-01	Conserved hypothetical protein.
Os06t0595900-02	Hypothetical conserved gene.
Os06t0596300-01	Similar to Acyl-ACP thioesterase
Os06t0597000-01	Similar to Auxin responsive protein IAA-Re.
Os06t0599700-00	Conserved hypothetical protein.
Os06t0612001-00	Conserved hypothetical protein.
Os06t0612900-00	Similar to predicted protein.
Os06t0613000-01	Hypothetical conserved gene.
Os06t0614300-01	C-CAP/cofactor C-like domain domain containing protein.
Os06t0619066-00	Hypothetical protein.
Os06t0619600-00	Hypothetical conserved gene.
Os06t0621500-01	Conserved hypothetical protein.
Os06t0623700-00	Similar to strictosidine synthase 1.
