# Supplementary material for: Mutations on ent-kaurene oxidase 1 encoding gene attenuate its enzyme activity of catalyzing the reaction from ent-kaurene to ent-kaurenoic acid and lead to delayed germination in rice
Source: PLoS Genet. 2020 Jan 10;16(1):e1008562. doi: 10.1371/journal.pgen.1008562 (PMC6977763; doi:10.1371/journal.pgen.1008562)
Supplement: S2 Table — (PDF) [file pgen.1008562.s002.pdf]

Table S2. Summary of RNA-seq data generated by Illumina Hiseq platform.

The m, wt and mGA stand for the mutant, wild-type and mutant treated with exogenous GA samples, respectively.

	mGA1	mGA2	mGA3	m1	m2	m3	wt1	wt2	wt3
**Total reads**	12890197	13205410	12762095	10403277	15443322	13607694	13607965	12781800	13405066
**High-quality reads**	12867509	13191829	12750361	10390339	15425777	13596087	13599479	12771426	13394964
**High-quality reads (%)**	99.82	99.90	99.91	99.87	99.89	99.91	99.94	99.92	99.92
**Low-quality reads**	22688	13581	11734	12938	17545	11607	8486	10338	10102
**Low-quality reads (%)**	0.18	0.10	0.09	0.13	0.11	0.09	0.06	0.08	0.08
**GC percentage (%)**	58.67	54.58	54.70	57.02	57.27	55.10	55.00	56.78	55.76
**Error rate (%)**	0.01	0.01	0.01	0.01	0.01	0.01	0.01	0.01	0.01
**Q20 (%)**	98.39	98.51	98.53	98.53	98.45	98.45	98.27	98.19	98.15
**Q30 (%)**	96.92	97.14	97.16	97.15	97.03	96.99	96.68	96.56	96.50
**Mapped reads**	12344862	12641277	12219629	9957675	14793553	13121038	13204902	12288772	12914953
**Mapped ratio (%)**	95.94	95.83	95.84	95.84	95.9	96.51	97.1	96.22	96.42
**FPKM >60**	2292	2427	2427	2261	2062	2139	2388	2110	2138
