# Supplementary material for: Mutations on ent-kaurene oxidase 1 encoding gene attenuate its enzyme activity of catalyzing the reaction from ent-kaurene to ent-kaurenoic acid and lead to delayed germination in rice
Source: PLoS Genet. 2020 Jan 10;16(1):e1008562. doi: 10.1371/journal.pgen.1008562 (PMC6977763; doi:10.1371/journal.pgen.1008562)
Supplement: S3 Table — (PDF) [file pgen.1008562.s003.pdf]

Table S3. Significantly differentially expressed genes.

Gene ID					description
a	$FPKM_{mGA}$	$FPKM_m$	$FPKM_{wt}$	$FPKM_m$	
OS01G0256500	111601.50	291251.10	207709.36	294198.	LEA-5
OS03G0159600	1819.93	8177.40	10056.83	8247.76	Seed maturation protein
OS03G0168100	2564.30	7673.76	9692.15	7749.36	LEA-3
OS03G0197300	14451.37	52231.60	50474.80	52702.8	Cupin 1
OS03G0663800	187.39	1862.15	747.42	1879.23	Cupin 1
OS03G0747400	308.49	2190.61	1208.32	2209.72	Seed maturation protein
OS03G0747500	307.35	1510.17	809.24	1523.56	Seed maturation protein
OS03G0793700	3811.01	38233.88	15852.14	38559.8	Cupin 1
OS04G0589800	5021.79	18148.51	15282.35	18317.9	LEA-25
OS08G0127900	3626.92	16969.55	7848.61	17121.9	11-S seed storage protein
OS12G0626500	103.30	1166.20	736.95	1175.43	Seed maturation protein
OS03G0342900	770.47	2671.60	1902.64	2697.52	Dormancy associated
OS03G0607200	480.89	2027.50	1041.83	2048.19	GA regulated protein
OS11G0671000	485.25	2198.98	995.56	2221.54	Dormancy associated
OS02G0186200	15.84	56.75	16.80	57.26	Cytochrome P450
OS03G0856700	1121.73	5239.61	2617.93	5298.03	GA20ox
OS06G0562200	72.99	347.76	153.49	350.82	PYR/PYL
OS10G0564500	179.77	740.14	455.90	746.66	SnRK2
OS06G0612800	8783.12	28685.08	17515.81	28950.2	Zinc finger
OS01G0702500	181.92	2957.40	2253.26	2980.62	Dehydrin
OS01G0762500	1391.53	2157.24	756.29	2182.47	11-S seed storage protein
OS02G0242600	31.30	91.69	6.86	92.48	11-S seed storage protein
OS02G0249000	141.35	192.49	62.89	194.64	11-S seed storage protein
OS02G0249600	1000.83	1676.17	347.41	1693.82	11-S seed storage protein
OS03G0427300	290.45	779.86	187.30	787.33	11-S seed storage protein
OS10G0400200	937.97	1408.66	548.97	1424.94	11-S seed storage protein
OS02G0453600	331.55	437.83	79.18	443.20	Cupin 1
b	$FPKM_{wt}$	$FPKM_m$	log_2Ratio	pval	
OS01G0762500	756.29	2182.47	-1.52	5.67E-05	11-S seed storage protein
OS02G0242600	6.86	92.48	-3.75	1.54E-05	11-S seed storage protein
OS02G0249000	62.89	194.64	-1.62	7.82E-05	11-S seed storage protein
OS02G0249600	347.41	1693.82	-2.28	2.14E-09	11-S seed storage protein
OS03G0427300	187.30	787.33	-2.07	2.88E-07	11-S seed storage protein
OS10G0400200	548.97	1424.94	-1.37	2.69E-04	11-S seed storage protein
OS02G0453600	79.18	443.20	-2.48	1.30E-08	Cupin 1
OS05G0328800	12.69	53.75	-2.08	1.36E-04	alpha-amylase inhibitor
OS05G0331532	6.38	31.65	-2.30	1.38E-04	alpha-amylase inhibitor
OS07G0219400	80.96	237.74	-1.55	1.58E-04	Bifunctional inhibitor
OS06G0569900	0	12.16	Infinite	1.61E-05	Cytochrome P450
c	$FPKM_{wt}$	$FPKM_m$	log_2Ratio	pval	

OS08G0338000	491.89	136.87	1.84	4.20E-06	Myb/SANT-like domain
OS11G0492800	9.28	0	Infinite	2.79E-04	Alpha/Beta hydrolase fold
OS11G0620300	314.16	23.71	3.72	2.34E-16	Bifunctional inhibitor
OS06G0204100	164.95	4.76	5.11	4.38E-20	Cytochrome P450
OS07G0217600	13.53	0.43	4.96	5.30E-05	Cytochrome P450
OS09G0325700	953.36	302.30	1.65	1.87E-05	PP2C
d	$FPKM_{mGA}$	$FPKM_m$	log_2Ratio	pval	
OS01G0256500	111601.50	291251.10	-1.38	4.03E-04	LEA-5
OS03G0159600	1819.93	8177.40	-2.16	6.31E-04	Seed maturation protein
OS03G0168100	2564.30	7673.76	-1.58	1.90E-04	LEA-3
OS03G0197300	14451.37	52231.60	-1.85	4.15E-04	Cupin 1
OS03G0663800	187.39	1862.15	-3.31	1.78E-08	Cupin 1
OS03G0747400	308.49	2190.61	-2.82	3.31E-05	Seed maturation protein
OS03G0747500	307.35	1510.17	-2.29	4.85E-04	Seed maturation protein
OS03G0793700	3811.01	38233.88	-3.32	1.13E-05	Cupin 1
OS04G0589800	5021.79	18148.51	-1.85	1.44E-04	LEA-25
OS08G0127900	3626.92	16969.55	-2.22	4.29E-05	11-S seed storage protein
OS12G0626500	103.30	1166.20	-3.49	2.38E-04	Seed maturation protein
OS03G0342900	770.47	2671.60	-1.79	1.28E-05	Dormancy associated
OS03G0607200	480.89	2027.50	-2.07	7.32E-07	GA regulated protein
OS11G0671000	485.25	2198.98	-2.18	8.20E-08	Dormancy associated
OS02G0186200	15.84	56.75	-1.84	3.62E-04	Cytochrome P450
OS03G0856700	1121.73	5239.61	-2.22	3.26E-07	GA20ox
OS06G0562200	72.99	347.76	-2.25	2.39E-04	PYR/PYL
OS10G0564500	179.77	740.14	-2.04	3.05E-04	SnRK2
OS01G0702500	181.92	2957.40	-4.02	1.20E-04	Dehydrin
OS06G0612800	8783.12	28685.08	-1.70	1.59E-04	Zinc finger
e	$FPKM_{mGA}$	$FPKM_m$	log_2Ratio	pval	
OS01G0285300	63.76	18.57	1.77	4.69E-04	SANT/Myb domain
OS12G0564100	97.47	25.19	1.95	2.67E-05	SANT/Myb domain
OS11G0128500	24.32	2.12	3.51	3.24E-06	SANT/Myb domain
OS03G0103100	383.49	79.17	2.27	9.22E-05	lipid transfer protein
OS10G0191100	41.79	7.73	2.43	1.27E-05	lipid transfer protein
OS10G0349300	1005.69	258.07	1.96	1.43E-06	lipid transfer protein
OS10G0349400	465.67	87.29	2.41	2.90E-05	lipid transfer protein
OS10G0552200	682.63	243.06	1.48	1.55E-04	lipid transfer protein
OS07G0106200	961.10	330.04	1.54	8.10E-05	Sugar transporter
OS11G0643800	345.80	99.82	1.79	1.95E-05	Sugar transporter
OS08G0475400	650.73	216.41	1.58	1.22E-04	Alpha hydrolase fold-3
OS11G0138900	23.04	4.23	2.44	3.58E-04	Alpha hydrolase fold
OS02G0596300	150.68	43.77	1.78	5.50E-04	Cytochrome P450
OS03G0225900	90.74	19.69	2.20	4.66E-04	Cytochrome P450
OS03G0367101	57.28	7.48	2.93	2.42E-07	Cytochrome P450
OS03G0760500	45.27	9.85	2.19	4.82E-05	Cytochrome P450

OS06G0110000	271.82	84.89	1.67	1.28E-04	Cytochrome P450
OS07G0603700	77.32	23.54	1.71	2.58E-04	Cytochrome P450
OS11G0289700	1334.61	295.90	2.17	3.74E-07	Cytochrome P450
OS12G0582700	67.86	19.84	1.77	4.91E-04	Cytochrome P450
OS01G0209700	32.74	7.49	2.12	5.58E-04	GA2ox

a: Genes have higher expression in mutant compared with both wild-type and mutant treated with GA treatment. b: Genes have higher expression in mutant only than wild-type. c: Genes have higher expression in wild-type compared to mutant. d: Genes have higher expression in mutant only than mutant with GA treatment. e: Genes have higher expression in mutant with GA compared to mutant. Gene IDs with red font were those also detected in proteomic study.
