# Supplementary material for: Mutations on ent-kaurene oxidase 1 encoding gene attenuate its enzyme activity of catalyzing the reaction from ent-kaurene to ent-kaurenoic acid and lead to delayed germination in rice
Source: PLoS Genet. 2020 Jan 10;16(1):e1008562. doi: 10.1371/journal.pgen.1008562 (PMC6977763; doi:10.1371/journal.pgen.1008562)
Supplement: S5 Table — (PDF) [file pgen.1008562.s005.pdf]

Table S5. Primers used in this study.

Primer name	Primer sequence	Purpose
CPS-F	5' GCGTGCATTTTCGAACCAA 3'	Real-time PCR
CPS-R	5' TTGGCCAGCACTGACACTCT 3'	Real-time PCR
KS-F	5' GTTCCCGTCAGCATCCGTG 3'	Real-time PCR
KS-R	5' GCTCCTGCCCAACATTCCA 3'	Real-time PCR
OsKO1-F	5' CCCGCCTCCAGCTGTTC 3'	Real-time PCR
OsKO1-R	5' AGCCCCGGTCCTTATAGTGT 3'	Real-time PCR
OsKO2-F	5' CTACAATGCTACTGTGACCGAC 3'	Real-time PCR
OsKO2-R	5' ATCCTTTCCTTCTGCTGCTT 3'	Real-time PCR
OsKO4-F	5' CATCCTGGCTGCAGCTGATA 3'	Real-time PCR
OsKO4-R	5' GGAACACGGCGTTGAGGTA 3'	Real-time PCR
KAO-F	5' CAGCAACGCAGAACGGATTA 3'	Real-time PCR
KAO-R	5' ACGGAAGGACACGAAGGAGA 3'	Real-time PCR
GA20ox1-F	5' TACTGCCACGAGATGAGCC 3'	Real-time PCR
GA20ox1-R	5' ACGCCGGGTAGTAGTTGAG 3'	Real-time PCR
GA20ox2-F	5' GACCGCTTCGCCTCCAA 3'	Real-time PCR
GA20ox2-R	5' GCGACAGCTCCTTCATCTCC 3'	Real-time PCR
GA3ox2-F	5' TCTTCTCCAAGCTCATGTGGT 3'	Real-time PCR
GA3ox2-R	5' AACTCCTCCATCACGTCACAG 3'	Real-time PCR
GA2ox1-F	5' CGAGCAAACGATGTGGAAGGGCTACAGG 3'	Real-time PCR
GA2ox1-R	5' TGGCTCAGGCGGAGTGAGTACATTGTCG 3'	Real-time PCR
OsNCED2-F	5' AGCCGACGATGATCCACGACTT 3'	Real-time PCR
OsNCED2-R	5' GCCTCCTCCCACGCATTCCACA 3'	Real-time PCR
OsNCED3-F	5' TCTTCGCGCTCAGCTACAATGT 3'	Real-time PCR
OsNCED3-R	5' GCCTTCTCCCTGTCGTATACCA 3'	Real-time PCR
OsABA8ox2-F	5' CGTCTTCTTCGCCTCCAA 3'	Real-time PCR
OsABA8ox2-R	5' ATCATCCGCTCCTTGCTC 3'	Real-time PCR
Amylase-2-F	5' ACTTCTTGTCCCTTTCCG 3'	Real-time PCR
Amylase-2-R	5' GCCCATAAGCAGGTTGTA 3'	Real-time PCR
GAMYB-F	5' GAATCCACCCCTCCTGTT 3'	Real-time PCR
GAMYB-R	5' GCCCCATTACTTGCTCTC 3'	Real-time PCR
GID2-F	5' TCGGTGTTTTATTACGAGATCC 3'	Real-time PCR
GID2-R	5' GAAGTCCACAGCGTCAGGTT 3'	Real-time PCR
ARAG1-F	5' CAACGACGGTGAGGGAGGAG 3'	Real-time PCR
ARAG1-R	5' CGGGGAACAGTAGCGAGCAG 3'	Real-time PCR
PYR/PYL-F	5' GGCGGTGTCGTGTTATTTGT 3'	Real-time PCR
PYR/PYL-R	5' CCGTACAAACCAAAACAGGCT 3'	Real-time PCR
PP2C-F	5' GTCACCCAGCTGATGCTGTA 3'	Real-time PCR
PP2C-R	5' TAGCAGTTGCACAACCCCAT 3'	Real-time PCR
SnRK2-F	5' GAGGGAGAAGGCCGACTACA 3'	Real-time PCR

SnRK2-R	5' TAGGTGTCATACTCATCGGCG 3'	Real-time PCR
U50-F	5' CGAGAACGGCGAGAAGTGGT 3'	Real-time PCR
U50-R	5' GGACGGAGATGGTATGGAGA 3'	Real-time PCR
U53-F	5' TGCTCAACCCCGTCTCCACT 3'	Real-time PCR
U53-R	5' TTGCTCAGCTCCCGTATCTG 3'	Real-time PCR
T46-F	5' CGAGAACTGCTACGGATGGA 3'	Real-time PCR
T46-R	5' CGGGATGCTGGGACAGACTA 3'	Real-time PCR
U49-F	5' AGATGGGGGAGGAAGGGT 3'	Real-time PCR
U49-R	5' CTCGTCGATGTCGATGCC 3'	Real-time PCR
U32-F	5' GCGACCAAGAACAAGCTGG 3'	Real-time PCR
U32-R	5' TCACGCCGTCCTTGAACTC 3'	Real-time PCR
U47-F	5' ATGAGCCAGGAGCAGCCGA 3'	Real-time PCR
U47-R	5' GTCTTCCCCAGCACGAGGT 3'	Real-time PCR
U51-F	5' GCGACGGAGAACATCTACG 3'	Real-time PCR
U51-R	5' GCCTTCACCCGCATCATCA 3'	Real-time PCR
U2-F	5' GGGCAGGTGATGGCGCAGTT 3'	Real-time PCR
U2-R	5' ACGGGCTCGTTGTCGTCGGA 3'	Real-time PCR
D30-F	5' AACGATCGGGAGACGGGGAG 3'	Real-time PCR
D30-R	5' GTTGACGGTGATGTTGCGGC 3'	Real-time PCR
R21-F	5' GCGTTCGTGACCTTGAAAAGCA 3'	Real-time PCR
R21-R	5' CGTTGGGGTCGTAGGAGCAGAG 3'	Real-time PCR
U15-F	5' CGGGTTAAGGCAATGGAGGT 3'	Real-time PCR
U15-R	5' TGTGGCGTTAGTTTGGGCAG 3'	Real-time PCR
T45-F	5' TCAACGACCCCTTCATCACC 3'	Real-time PCR
T45-R	5' AACCTTCTTGGCACCACCCT 3'	Real-time PCR
Osactin1-F	5' GGAAGTACAGTGTCTGGATTGGAG 3'	Real-time PCR
Osactin1-R	5' TCTTGGCTTAGCATTCTTGGGT 3'	Real-time PCR
Os06g0570100-F	5' ATGGAGGCGTTCGTGCCG 3'	CDS clone
Os06g0570100-R	5' TCACATCCTTCCTCTGCGCG 3'	CDS clone
Os06g0569900- F	5' ATGGAGTCGCTGCTCGCA 3'	CDS clone
Os06g0569900-R	5' TCACATCCTTCCTCTGGGC 3'	CDS clone
Os06g0569500-F	5' ATGGAGTCGATGCTCGTAGC 3'	CDS clone
Os06g0569500-R	5' TCACATCCTTCCTCTGGGCT 3'	CDS clone
Os06g0570100-promoter-F	5' ATCAATGCTCCTGGTTCTTTTG 3'	Promoter clone
Os06g0570100-promoter-R	5' GGATCACACGCTGTGCGC 3'	Promoter clone
Os06g0569900-promoter-F	5' GAAAATGTTTCACTCATTTAAATCA 3'	Promoter clone
Os06g0569900-promoter-R	5' GGCCGACCCCTACTCAGA 3'	Promoter clone
Os06g0569900RNA	5'GGGGACAAGTTTGTACAAAAAAGCAGGCTT	OsKO1 RNAi

i-F	CCCTCAATGCCGTCTTCCAG 3'	
Os06g569900RNAi-R	5'GGGGACCACTTTGTACAAGAAAGCTGGGTCTTCTCCTCGTCACCCTCCCTCA 3'	OsKO1 RNAi
Os06g0570100RNAi-F	5' GGGGACAAGTTTGTACAAAAAAGCAGGCTTCACCTCAACGCCGTCTTCCA 3'	OsKO2 RNAi
Os06g0570100RNAi-R	5' GGGGACCACTTTGTACAAGAAAGCTGGGTCTTCTCCTCGTCACCCTCCCT 3'	OsKO2 RNAi
KO2-taqman-F	5' ATGAACAGGAAGGAGTGGGAGT 3'	Linkage analysis
KO2-taqman-R	5' TGGAGCTTGTAGGCGGTGAG 3'	Linkage analysis
