# Supplementary material for: Mutations on ent-kaurene oxidase 1 encoding gene attenuate its enzyme activity of catalyzing the reaction from ent-kaurene to ent-kaurenoic acid and lead to delayed germination in rice
Source: PLoS Genet. 2020 Jan 10;16(1):e1008562. doi: 10.1371/journal.pgen.1008562 (PMC6977763; doi:10.1371/journal.pgen.1008562)

Fig. S1. Distribution and sequence alignment analyses of *OsKO* genes. (A) Contiguously arranged five *OsKO* genes in a 120 kb highly linked region on the sixth chromosome of rice genome. The row marked by red filled circle shows the old nomenclature of *OsKO genes*, and that marked by green filled circle shows the updated name of each *OsKO gene*. (B) Three-dimensional structures of the wild type (left panel) and mutated (right panel) OsKO1 proteins. The green arrow indicates the mutated site.

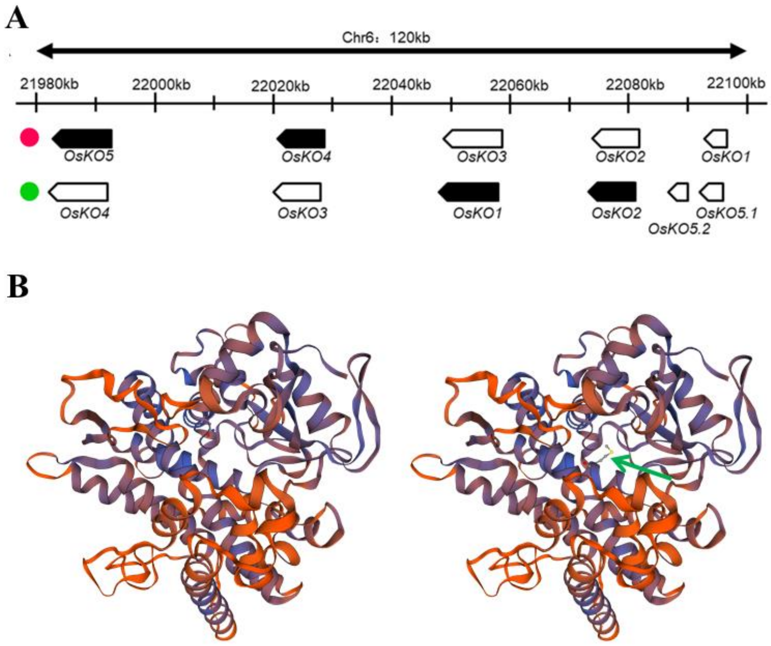

Supplement: S1 Fig — (A) Contiguously arranged five OsKO genes in a 120 kb highly linked region on the sixth chromosome of rice genome. The row marked by red filled circle shows the old nomenclature of OsKO genes, and that marked by green filled circle shows the updated name of each OsKO gene. (B) Three-dimensional structures of the wild type (left panel) and mutated (right panel) OsKO1 proteins. The green arrow indicates the mutated site. (PDF) [file pgen.1008562.s006.pdf]
