# Supplementary material for: Mutations on ent-kaurene oxidase 1 encoding gene attenuate its enzyme activity of catalyzing the reaction from ent-kaurene to ent-kaurenoic acid and lead to delayed germination in rice
Source: PLoS Genet. 2020 Jan 10;16(1):e1008562. doi: 10.1371/journal.pgen.1008562 (PMC6977763; doi:10.1371/journal.pgen.1008562)

Fig. S2. Sequence alignment analysis of KO proteins from rice, Arabidopsis, wheat and Nelumbo nucifera. The read rectangle and arrow indicate the mutated site.

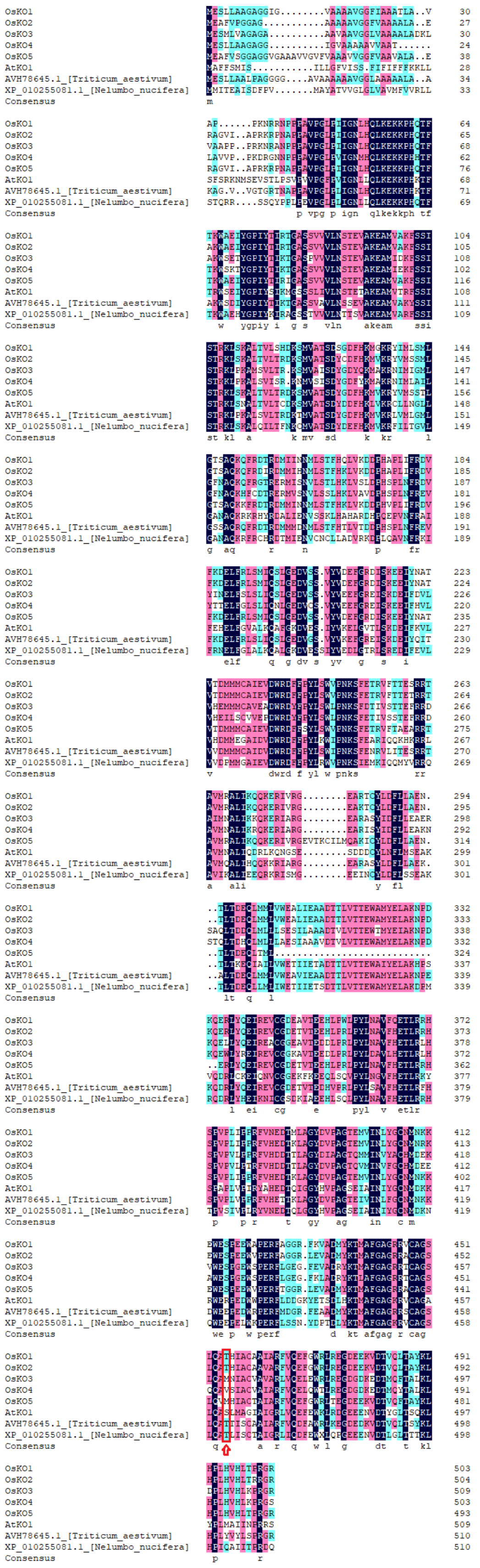

Supplement: S2 Fig — The read rectangle and arrow indicate the mutated site. (PDF) [file pgen.1008562.s007.pdf]
