# Supplementary material for: Mutations on ent-kaurene oxidase 1 encoding gene attenuate its enzyme activity of catalyzing the reaction from ent-kaurene to ent-kaurenoic acid and lead to delayed germination in rice
Source: PLoS Genet. 2020 Jan 10;16(1):e1008562. doi: 10.1371/journal.pgen.1008562 (PMC6977763; doi:10.1371/journal.pgen.1008562)

Fig. S3. A) Chromatograph and mass spectra of ent-kaurene, ent-kaurenol, and ent-kaurenoic acid standards. The three panels at left side are the chromatograph images, and those at the right side are the corresponding mass spectra for each chemical. B) GC-MS analysis on the amounts of intermediate product *ent*-kaurenol (peak 1) and final product *ent*-kaurenoic acid (peak 2) catalyzed by wild type (left panels) and mutated OsKO1 (right panels) from *ent*-kaurene at 10 (up panels) and 20 min (bottom panels).

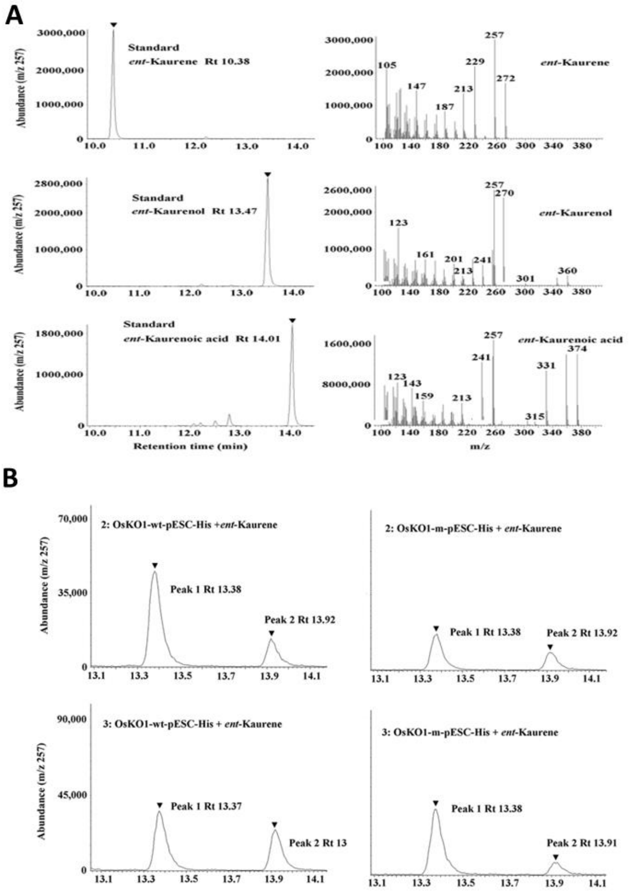

Supplement: S3 Fig — (A) Chromatograph and mass spectra of ent-kaurene, ent-kaurenol, and ent-kaurenoic acid standards. The three panels at left side are the chromatograph images, and those at the right side are the corresponding mass spectra for each chemical. (B) GC-MS analysis on the amounts of intermediate product ent-kaurenol (peak 1) and final product ent-kaurenoic acid (peak 2) catalyzed by wild type (left panels) and mutated OsKO1 (right panels) from ent-kaurene at 10 (up panels) and 20 min (bottom panels). (PDF) [file pgen.1008562.s008.pdf]
