# Supplementary material for: Mutations on ent-kaurene oxidase 1 encoding gene attenuate its enzyme activity of catalyzing the reaction from ent-kaurene to ent-kaurenoic acid and lead to delayed germination in rice
Source: PLoS Genet. 2020 Jan 10;16(1):e1008562. doi: 10.1371/journal.pgen.1008562 (PMC6977763; doi:10.1371/journal.pgen.1008562)

Fig. S4. Subcellular localization analysis of OsKO1 and OsKO2 with their full length CDS fused with GFP protein. The GFP containing vector was used as control. Scale bars = 2.5 μm.

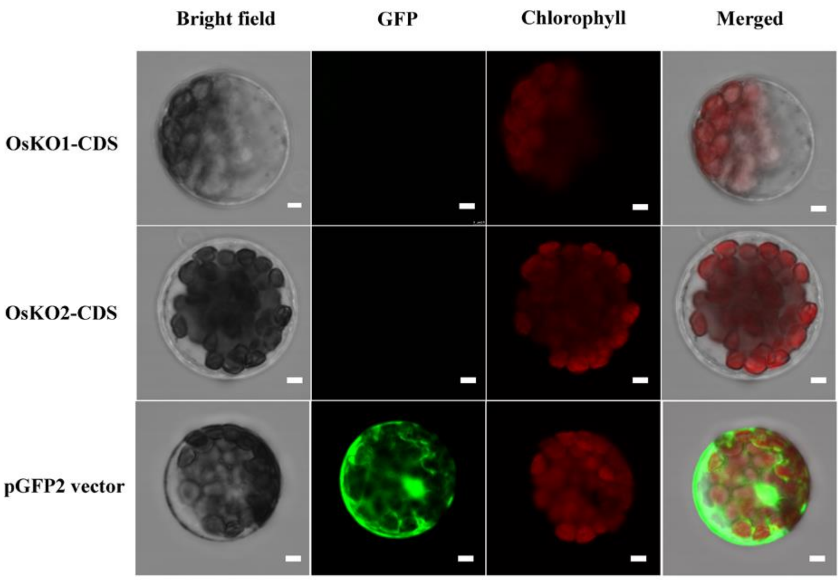

Supplement: S4 Fig — The GFP containing vector was used as control. Scale bars = 2.5 μm. (PDF) [file pgen.1008562.s009.pdf]
