# Supplementary material for: Mutations on ent-kaurene oxidase 1 encoding gene attenuate its enzyme activity of catalyzing the reaction from ent-kaurene to ent-kaurenoic acid and lead to delayed germination in rice
Source: PLoS Genet. 2020 Jan 10;16(1):e1008562. doi: 10.1371/journal.pgen.1008562 (PMC6977763; doi:10.1371/journal.pgen.1008562)

Fig. S5. RNA-seq and 2-DE analyses on the mutant and wild type rice. (A) Volcano plots showing the differentially expressed genes between wt and m (upside image), and m and m+GA (bottom image). mGA stands for m+GA. **(B)** Verification of the RNA-seq results through qRT-PCR on the selected genes in GA biosynthesis and ABA signaling pathways. (C) 2-D gel images of wt and m embryos. U and D indicate the up- and down-regulated proteins in mutant, respectively.

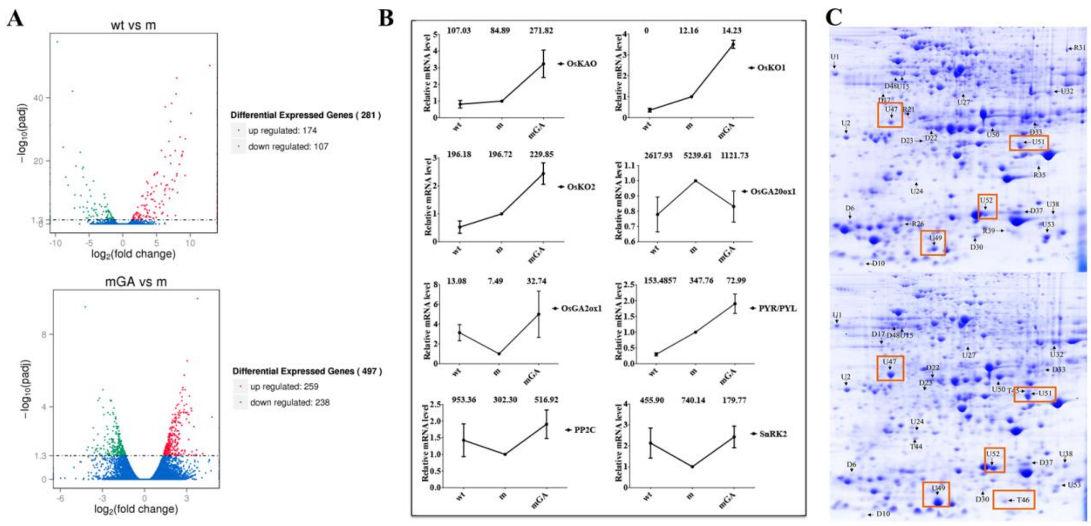

Supplement: S5 Fig — (A) Volcano plots showing the differentially expressed genes between wt and m (upside image), and m and m+GA (bottom image). mGA stands for m+GA. (B) Verification of the RNA-seq results through qRT-PCR on the selected genes in GA biosynthesis and ABA signaling pathways. (C) 2-D gel images of wt and m embryos. U and D indicate the up- and down-regulated proteins in mutant, respectively. (PDF) [file pgen.1008562.s010.pdf]
